# Cutaneous Angiomyolipoma of the Forearm

**DOI:** 10.7759/cureus.103101

**Published:** 2026-02-06

**Authors:** Nor Haizura Abd Rani, Lia Natasha, Ni Tee San, Phraveender Kaur Sandhu, Fairrul Kadir

**Affiliations:** 1 Department of Pathology, Universiti Malaysia Sabah, Kota Kinabalu, MYS; 2 Department of Pathology and Microbiology, Faculty of Medicine and Health Sciences, Universiti Malaysia Sabah, Kota Kinabalu, MYS; 3 Department of Diagnostic Laboratory Services, Hospital Canselor Tuanku Muhriz UKM (Universiti Kebangsaan Malaysia), Cheras, MYS; 4 Department of Pathology, Queen Elizabeth Hospital, Kota Kinabalu, MYS; 5 Department of Emergency Medicine, Faculty of Medicine and Health Sciences, Universiti Malaysia Sabah, Kota Kinabalu, MYS

**Keywords:** angiomyolipoma, benign tumour, cutaneous, histopathology, immunohistochemistry

## Abstract

Angiomyolipoma (AML) is defined as a benign tumour that is commonly identified in the kidneys, and most cases are associated with tuberous sclerosis. Although extrarenal AML cases have been reported, the association with the liver remains the most prevalent. We hereby report a rare case of cutaneous AML on the forearm of a 62-year-old gentleman, enduring for 10 years. Histopathology of AML revealed a combination of mature adipose tissue, thick-walled blood vessels, and smooth muscle cells, which distinguishes AML from other tissue tumours, such as lipoma, angiolipoma, hemangioma, and other mixed mesenchymal neoplasms. Immunochemistry was negative for human melanoma black-45 (HMB-45), emphasising the diagnosis of cutaneous AML and excluding it from renal and extrarenal AML. Cutaneous AML is a well-circumscribed, solitary, and non-invasive tumour that can be completely removed via surgery without recurrence. We want to highlight the importance of histopathology and immunohistochemistry evaluation in identifying cutaneous AML. We report a case of a 62-year-old gentleman who presented with a painless and gradually enlarging single subcutaneous swelling on his left forearm for a duration of 10 years.

## Introduction

Angiomyolipoma (AML) is a benign mesenchymal neoplasm composed of a characteristic triad of mature adipose tissue, thick-walled blood vessels, and bundles of smooth muscle cells [1]. These distinctive histological features allow AML to be differentiated from other soft tissue tumours such as lipoma, angiolipoma, hemangioma, and other mixed mesenchymal neoplasms [2].

More than 99% of AMLs arise in the kidney, with approximately one-third of cases associated with tuberous sclerosis complex [3]. Extrarenal AMLs are uncommon and have been reported in various anatomical locations, including the liver, nasal cavity, oral cavity, heart, colon, lung, and skin [3]. Renal AMLs are typically invasive, composed of perivascular epithelioid cells, express human melanoma black-45 (HMB-45), and predominantly occur in female patients [4].

Cutaneous AML is a rare subtype of extrarenal AML. Unlike its renal counterpart, cutaneous AML usually presents as a solitary, well-circumscribed, and non-invasive lesion, occurs more frequently in males, lacks an association with tuberous sclerosis, and is consistently negative for HMB-45 immunostaining [4]. Due to its rarity and nonspecific clinical presentation, cutaneous AML is often misdiagnosed as more common benign soft tissue lesions. Accurate diagnosis, therefore, relies on careful histopathological and immunohistochemical evaluation.

## Case presentation

A 62-year-old male with no significant past medical or surgical history presented with a single, painless, and gradually enlarging subcutaneous swelling on the lateral aspect of his left forearm for 10 years. The swelling did not interfere with arm movement. There was no history of bleeding, trauma, or infection. No other swellings were noted, and there were no associated systemic symptoms.

On examination, the swelling was firm, well-circumscribed, non-erythematous, mobile, and measured approximately 4.0 × 3.0 cm. There were no signs of tuberous sclerosis or infection.

A forearm lipoma was clinically suspected. The lesion was completely excised under local anaesthesia in a day-care procedure without complication. No imaging or laboratory tests were performed before or after excision. Macroscopically, the mass measured 15.0 × 15.0 × 7.0 mm, was firm, globular, and well-circumscribed. Serial cut sections revealed a homogenous whitish-yellow surface.

**Figure 1:**
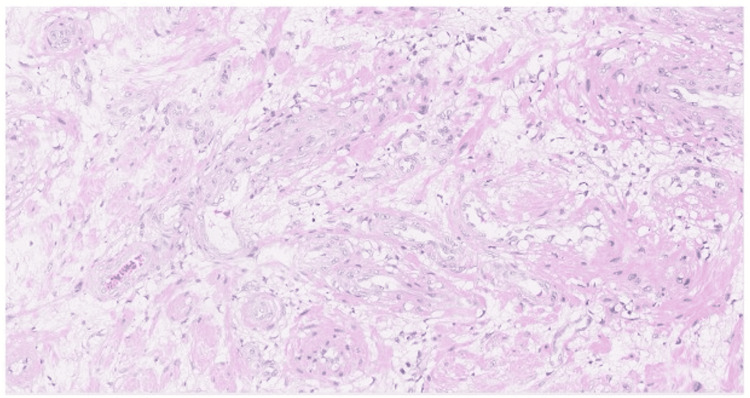
Histopathological examination showing mature adipose tissue, thick-walled blood vessels, and smooth muscle components (hematoxylin and eosin stain, ×10).

Microscopy demonstrated a well-circumscribed lesion composed of various-sized thick-walled blood vessels lined by plump endothelial cells (Figure 1). These vessels were surrounded by bundles of smooth muscle cells arranged in interlacing fascicles. Interspersed mature adipocytes were also observed. No malignant features were present.

Immunohistochemistry using the Ventana BenchMark ULTRA platform with UltraView detection system (Roche, Basel, Switzerland) showed positive staining for actin and CD31, while monoclonal antibody HMB-45 and S100 were negative. Both positive and negative controls were adequate. Based on histology and immunoprofile, a diagnosis of cutaneous angiomyolipoma was confirmed.

## Discussion

Cutaneous AML, also referred to as cutaneous angiolipoleiomyoma, is an uncommon extrarenal form of AML. It was first described in 1990 by Fitzpatrick et al. [5]. To date, only 44 cases of cutaneous AML have been reported, as reviewed by Sanchez et al. [6].

Cutaneous AML predominantly occurs in middle-aged males and is not associated with tuberous sclerosis [5]. Clinically, lesions are solitary, asymptomatic, well-demarcated, and non-invasive, often being misdiagnosed as more common soft tissue lesions such as lipoma, fibrolipoma, or mucoid cyst [3,5].

Histologically, cutaneous AML exhibits the classic triad of mature adipose tissue, thick-walled blood vessels, and smooth muscle cells, often enclosed within a well-circumscribed fibrous pseudocapsule [4]. Unlike renal AML, cutaneous AML is negative for HMB-45 immunostaining, as confirmed in our case and reported in previous studies [3-6].

Cutaneous AML is benign, does not progress, and recurrence is typically due to incomplete excision [6]. Complete surgical excision is therefore the treatment of choice and is generally curative [4].

Learning points

Cutaneous AML is a rare, benign mesenchymal tumour that should be considered in the differential diagnosis of long-standing, painless subcutaneous swellings. Histopathology demonstrating the triad of mature adipose tissue, thick-walled blood vessels, and smooth muscle cells is essential for diagnosis. Immunohistochemistry showing HMB-45 negativity differentiates cutaneous AML from renal and other extrarenal AMLs. Complete surgical excision is curative with minimal risk of recurrence.

## Conclusions

In contrast to renal AML, cutaneous AML is solitary, well-demarcated, non-invasive, not associated with tuberous sclerosis, and effectively treated with simple surgical excision. These findings are consistent with previously reported literature. Cutaneous AML should be considered in the differential diagnosis of forearm swellings, particularly when histopathology demonstrates the classic triad and immunohistochemistry is negative for HMB-45. Complete excision ensures an excellent prognosis.
